# Separation of 9-Fluorenylmethyloxycarbonyl Amino Acid Derivatives in Micellar Systems of High-Performance Thin-Layer Chromatography and Pressurized Planar Electrochromatography

**DOI:** 10.1038/s41598-019-53468-9

**Published:** 2019-11-19

**Authors:** Beata Polak, Adam Traczuk, Sylwia Misztal

**Affiliations:** Department of Physical Chemistry, Medical University of Lublin, Chodźki 4a, 20-093 Lublin, Poland

**Keywords:** Analytical chemistry, Biochemistry

## Abstract

The problems with separation of amino acid mixtures in reversed-phase mode are the result of their hydrophilic nature. The derivatisation of the amino group of mentioned above solutes leads to their solution. For this purpose, 9-fluorenylmethoxycarbonyl chloroformate (f-moc-Cl) as the derivatisation reagent is often used. In our study, the separation of some f-moc- amino acid derivatives (alanine, phenylalanine, leucine, methionine, proline and tryptophan) with the use of micellar systems of reversed-phase high-performance thin-layer chromatography (HPTLC) and pressurized planar electrochromatography (PPEC) is investigated. The effect of surfactant concentration, its type (anionic, cationic and non-ionic) and mobile phase buffer pH on the discussed above solute migration distances are presented. Our work reveals that the increase of sodium dodecylsulphate concentration in the mobile phase has a different effect on solute retention in HPTLC and PPEC. Moreover, it also affects the order of solutes in both techniques. In PPEC, in contrast to the HPTLC technique, the mobile phase pH affects solute retention. The type of surfactant in the mobile phase also impacts solute retention and migration distances. A mobile phase containing SDS improves system efficiency in both techniques. Herein, such an effect is presented for the first time.

## Introduction

Amino acids contain two functional ionisable (carboxylic and amine) groups. Depending on their location, various classes of these compounds are distinguished i.e. α, β and γ. Amino acids may also bear a variety of entities such as: hydroxyl (-OH), imine (-NH) or sulphydryl (-SH) functional groups, aromatic rings or heterocyclic systems. Regarding the nature of the side chain, amino acids are non-polar (e.g. alanine), polar without charge (e.g. cysteine), acidic (e.g. aspartic acid) and basic (e.g. lysine). The most important amino acid reaction is the formation of a peptide bond between the amino group of one amino acid and the carboxyl group of another. Therefore, amino acids are the basic building blocks of peptides and proteins. The level of some amino acids in tissues may be also a marker of many different diseases^1–6^.

Since amino acids are high polar compounds and their detection requires special equipment (e.g. mass spectrometry), various reagents are used for pre-column derivatization. Of these, 9-fluorenyl-methyloxycarbonyl (f-moc) chloroformate is a popular derivatising reagent. It reacts almost immediately under mild conditions, both with primary and secondary amine groups of amino acid, and it yields stable compounds. The critical protocol on the derivatisation process has been presented in 2009^7^ and updated in 2011^8^. The introducing of f-moc moiety into a molecule enhances the hydrophobicity of solute and allows for their detection by way of UV-Vis spectrum,as well as fluorescence. The majority of f-moc amino acid derivative separations come about through high-performance liquid chromatography (HPLC) in the reversed phase mode, and the quantitative analysis of 20 amino acid f-moc derivatives has been presented in^9^. The validated method was applied for the analysis of amino acid content in *Isatis indigotica* (Fort) of various Chinese origins. Such investigations allowed for identification of where this herb was picked. The same kind of amino acid derivatives has been used for the simultaneous analysis of 13 amino acids in honey samples in^10^. A linear mobile phase gradient of acetonitrile and aqueous trifluoroacetic acid solution as components of the mobile phase was used for this purpose. The separation of 35 amines and amino acids without derivatisation in the polar stationary phase and after derivatisation with f-moc chloride and an RP-18 stationary phase has been compared in^11^. A mass spectrometry detector was used in both systems. This mode allied with the derivatisation process is more sensitive and achieves better results. This approach has also been applied for the analysis of amino acids in plasma samples.

The above presented examples of separation of amino acid derivatives involved the HPLC technique. Separation utilizing thin-layer chromatography (TLC) is not so common. Indeed, there is no literature data on separation of f-moc amino acid derivatives with the use of the latter technique. With regard to other amino acid derivatives, separation of phenylthiocarbamyl utilizing TLC was presented in^12^. These experiments were performed in both normal and reversed phase mode, while solute spot detection involved an *in-situ* iodine–azide reaction. More information can be found on non-derivatized amino acid separations. Herein, coupling ofthin-layer chromatography and pressurized planar electrochromatography intoa 2D system enhanced the separation of the non-derivatized amino acid^13^. Furthermore, amino acid separations with the use of pressurized planar electrochromatography (PPEC) and TLC techniques and silica gel as the stationary phase and acetonitrile-aqueous buffer mixture as the eluent was compared in^14^.

With regard to TLC and micellar eluent, Cerhati *et al*.^15^ investigated the effect of a methanol-aqueous mobile phase containing surfactant (SDS or non-ionic Genapol O 80) on the retention of amino acids and a homologous series of peptides. In their work, the stationary phase was specially prepared using aluminium oxide impregnated with a n-hexane paraffin oil mixture. They noticed that the presence of surfactant in the mobile phase affected strength interaction with stationary phase. In other studies, an eluent with a mixture of surfactants (SDS and tridecylalcohol diglycolate) of various concentrations has been applied for investigating low molecular mass peptide interactions with a stationary phase of the RP-type (aluminium oxide impregnated with n-hexane paraffin oil mixture)^16^.

In our study, we applied micellar systems of high-performance thin-layer chromatography (HPTLC) and PPEC to separate f-moc amino acid derivatives. To our knowledge, this was the first such exploration, as the application of pressurized planar electrochromatography with micellar eluent is not common. We also carried out a comparison of f-moc amino acid derivative behaviours in both modes (HPTLC and PPEC). The effect of surfactant concentration, its type, the mobile phase buffer pH on the solute retentions and migration distances was considered. What is more, we compared chromatographic parameters such as peak asymmetry and tailing factors, separation efficiency in both techniques (HPTLC and PPEC) with the mobile phases with or without surfactant. For PPEC, these parameters are the first such to be calculated.

## Results and Discussion

### Effect of surfactant concentration

**Figure 1:**
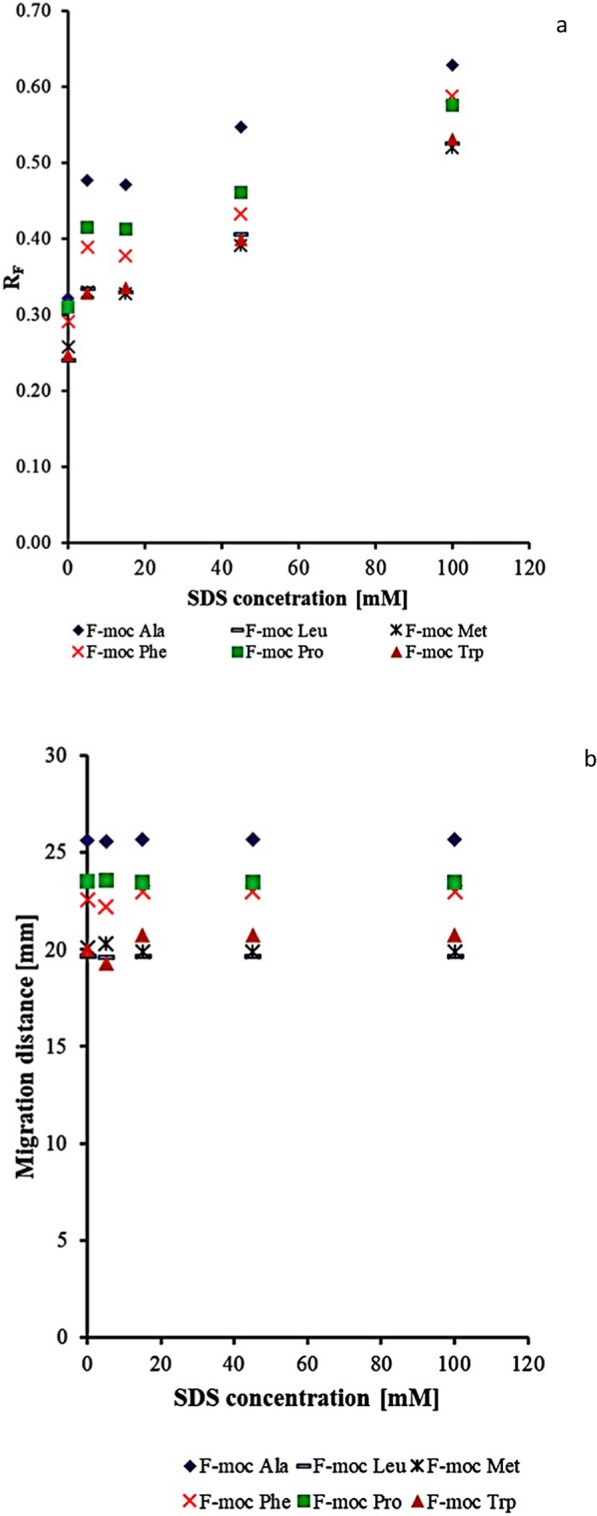
The effect of concentration of sodium dodecylsulphate in the mobile phase on the retention (HPTLC technique, (**a**)) and migration distances of f-moc amino acid derivatives (PPEC technique (**b**)). The mobile phase: acetonitrile (45%) and an aqueous acetic buffer of pH 4.25 (acetic acid and sodium acetate 7 and 3 mM, respectively). The stationary phase: HPTLC RP-18W. TLC experiment time − 15 min; PPEC polarization voltage 800 V, experiment time − 8 min.

The effect of surfactant concentration in the mobile phase on the retention (HPTLC) or migration distance (PPEC) of f-moc amino acids is presented in Fig. 1a,b. In the chromatographic system, the presence of surfactant (Fig. 1a) affects several processes. The first is the interaction between solute, micelles and components of the eluent. The second is the activity between micelle and the stationary phase. This induces changes in the physicochemical character. With regard to the first process, the presence of surfactant in the mobile phase influences the formation of solute-micelle complexes within this part of the chromatographic system. Thus, depending on the surfactant concentration in the eluent, the solute is either dissolved in the interfacial part of the micelle at low surfactant level or it penetrates the micelle at high amphiphile content^17^. Hence, the interactions with the stationary phase affect the total separation process. Thus, the retention of solutes depends on the strength of their bonding in the stationary phase and their rate of dissolving in the mobile phase. It should be noted that in thin-layer chromatography, the micelle mobile phase can affect the demixing of eluent during plate development, resulting in two mobile phase fronts. In our experiment, the higher of these held a higher content of water-organic (aqueous-acetonitrile) solvent, whereas a higher concentration of surfactant was present in the lower phase front. What is more, in the lower part of the plate, the mobile phase was more viscous than in the higher. As the result of such behaviour, the eluotropic strength of the mobile phase differed below and above the first (lower) eluent fronts. This effect was described in^18^.

Regarding the HPTLC technique (Fig. 1a), adding SDS to the mobile phase reduces solute retention of f-moc amino acids. Such behaviour is the result of the surfactant, dissociated f-moc derivatives and silanol groups from the stationary phase holding identical charges. In the stationary phase system, the interactions between the non-polar part of the SDS molecules and the alkyl chain of C-18 sorbent are hydrophobic and result in the increasing polarity of the sorbent. Thus, the solute associated with the SDS micelle (solubilised in its interfacial part) and the change of sorbent character deteriorates retention. Herein, the smallest retention is observed at 100 mM of SDS in the mobile phase. Nevertheless, this fact is not associated with improvement of solute zones separations. Thus, the best zone separation was observed when 45 mM of SDS was present in the eluent. We also discovered that the surfactant concentration affected the compound retention order. Furthermore, this varied for some f-moc derivatives at 5 and at 15 mM of SDS. According to our results, the migration order of the f-moc derivatives for 45 mM SDS starting from the compound of the strongest retention are as follows: f-moc Met < f-moc Trp < f-moc Leu < f-moc Phe < f-moc Pro < f-moc Ala.

In the PPEC technique (Fig. 1b), contrary to HPTLC, we found that the increase of SDS concentration in the mobile phase slightly increased the migration distances. This effect results from the opposing direction of electrophoretic migration of the micelle-solute complex relative to the electroosmotic flow. Similarly to TLC, the best selectivity was obtained for 45 mM of SDS in the mobile phase.The sequence of solute migration distances starting from the shortest for this eluent composition is as follows: f-moc Leu < f-moc Met < f-moc Trp < f-moc Phe < f-moc Pro < f -moc Ala. This order for the majority of solutes (exception is f-moc Met) is consistent with the decreasing pK_A_ values (see Table 1), but for some solutes, differs from TLC. Due to the process of pre-wetting the plate with the mobile phase, the phenomenon of two eluent fronts being seen, as in the electrochromatogram, was not observed. This phenomenon seems to confirm superior PPEC over TLC technique for systems containing surfactants.

**Table 1:** List of f-moc derivatives of amino acid derivatives and some of their physicochemical properties.

Lp.	Name	Producer	Symbol	Formula	pK_A_	logP MW (g/mol)
**Aliphatic amino acid derivatives**						
1.	f-moc alanine	Sigma-Aldrich, Germany	f-moc Ala		3.74	3.05 311.33
2.	f-moc Leucine	Sigma-Aldrich, Germany	f-moc Leu		3.91	4.30 353.41
3.	f-moc Methionine	Sigma-Aldrich, Germany	f-moc Met		3.82	3.70 371.45
**Aromatic amino acid derivatives**						
4.	f-moc Phenylalanine	Sigma-Aldrich, Germany	f-moc Phe		3.85	4.70 387.43
5.	f-moc Proline	Sigma-Aldrich, Germany	f-moc Pro		3.75	3.32 337.37
6.	f-moc Tryptophan	Sigma-Aldrich, Germany	f-moc Trp		3.92	4.80 426.47

The pKa-, logP and MW of each compound were drawn utilizing MarvinSketch^28^, formulas of amino acids originated from merckmilipore^29^.

### Effect of buffer pH

Since the interactions between investigated solutes, surfactant, stationary and mobile phases depend on ionization, we investigated the effect of mobile phase buffer pH on retention/migration distances. Two buffers of different pH were chosen for this purpose. The application of the first (pH = 2.5) resulted in the withdrawal of solute ionization. Thus, at this condition, interactions between neutral (hydrophobic) solute, surfactant and both stationary and mobile phases were made evident. The second buffer pH (7.0) is above the solute pK_A_ (see Table 1). At such pH, the solutes are ionized, hence, their hydrophobicity decreased. The effect of the mobile phase buffer pH on the f-moc amino acid derivative migration distances in both techniques is presented in Table 2.

**Table 2:** Comparison of retardation factors (HPTLC) and migration distances (PPEC) of f-moc amino acids at different mobile phase buffer pH.

Solute	SDS			
	R_F_ (HPTLC)		Migration distance (mm, PPEC)	
	pH 2.50	pH 7.00	pH 2.50	pH 7.00
f-moc Ala	0.43	0.36	19.6	22.8
f-moc Leu	0.26	0.27	16.9	18.8
f-moc Met	0.29	0.30	18.7	19.4
f-moc Phe	0.24	0.26	16.9	17.6
f-moc Pro	0.29	0.33	18.2	19.3
f-mocTrp	0.24	0.26	15.9	16.9

The mobile phase composition: 40% acetonitrile, 15 mM of SDS, 5% v/v universal buffer, 55% redistilled water; for the PPEC technique: polarization voltage 1.0 kV, experiment time − 10 min.

In TLC, the mobile phase buffer pH had insignificant influence on the retention of the majority of derivatives. Such behaviour is the result of the strong interactions between f-moc derivatives and the stationary phase being changed by surfactant addition. In comparison with the remaining solutes, f-moc Ala exhibited higher R_F_. This results from the effect of the weaker interactions with sorbent that comes about due to its aliphatic, more hydrophilic character and weaker dispersion forces (see log P, MW in Table 1). In our experiment, aromatic and more hydrophobic f-moc Phe and Trp showed the strongest retention (see Table 1). Application of the lowest pH buffer in the mobile phase, herein, led to poor resolution of f-moc Met and f-moc Pro zones, as well as f-moc Phe and f-moc Trp spots. In contrast, for the higher pH buffer, only two f-moc derivatives (f-moc Phe and f-moc Trp) did not separate.

On using PPEC as the experiment technique, we noted that the mobile phase buffer pH had a significant effect on the migration distances of the investigated f-moc derivatives. Herein, the hydrophobic effect between neutral solute (at pH 2.5), the hydrophobic interior of SDS micelle and the stationary phase resulted in stronger interactions with the stationary phase. Similarly to TLC experiments, the physicochemical character of derivatives affects the length of their migration distances (f-moc Ala has the longest, f-moc Trp the shortest). We also saw poor f-moc-Phe and f-moc Leu zone separation. This came about due to the increase of buffer pH to 7.0 resulting in the ionization of f-moc derivatives and enhancement of their hydrophilicity. Such effect led to stronger interactions in the mobile phase and the elongation of migration distances. Electroosmotic flow (EOF) also contributed to the solute migration distance length in PPEC. Furthermore, we saw a stronger EOF at higher pH - as also noted by^19^. In addition, an increase the mobile phase buffer pH improved the separation of f-moc Phe and f-moc Leu, but worsened the separation of f-moc Met and f-moc Pro.

### Comparison of surfactants

We also investigated the effect of surfactant type (cationic, anionic, non-ionic) on solute retention, migration distances and selectivity. Tests were carried out using both techniques (TLC and PPEC). We established the composition of the mobile phase on the basis of previous experiments^20^. This was comprised of 45% acetonitrile in an aqueous buffer of pH 2.5. Such a buffer pH allows an investigation of the neutral form of f-moc derivatives (the solutes pK_A_ are presented in Table 1). The following surfactants were applied during these experiments: Brij-35 (CMC in aqueous solution 0.1 mM), sodium cholate (CMC in aqueous solution 13–15 mM), hexadecyltrimethylammonium bromide (CTAB, CMC in aqueous solution 0.92 mM), sodium dodecyl sulphate (SDS, CMC in aqueous solution 8.1 mM) and tetramethylammonium chloride (CTMA, lack data of CMC in aqueous solution). Since both organic solvent (acetonitrile) and pH affects the surfactant CMC value, to be sure that the chosen micellar systems were applied, high concentrations of investigated surfactants were prepared. In this experiment, experiment time for HPTLC was 15 min, while for PPEC,the corresponding time was only 10 min (when the polarization voltage was 800 V). The results are presented in Table 3.

**Table 3:** Comparison of f-moc derivative retardation factors (HPTLC) and migration distances (PPEC) of investigated f-moc derivatives for various surfactant systems.

	Retardation factors (HPTLC technique)				
	Non-ionic	Anionic		Cationic	
	Brij-35 40 mM	Sodium cholate 45 mM	SDS 60 mM	CTMA 45 mM	CTAB 75 mM
f-moc Ala	0.41	0.44	0.45	0.53	0.38
f-mocPhe	0.51	0.40	0.32	0.41	0.28
f-moc Leu	0.53	0.40	0.35	0.40	0.29
f-moc Met	0.43	0.42	0.40	0.48	0.32
f-moc Pro	0.52	0.40	0.40	0.46	0.35
f-mocTrp	0.44	0.40	0.33	0.41	0.27
**Solute**	**Migration distances in mm (PPEC technique)**				
	**Non-ionic**	**Anionic**		**Cationic**	
	**Brij-35 40 mM**	**Sodium cholate 45 mM**	**SDS 60 mM**	**CTMA 45 mM**	**CTAB 75** **mM**
f-moc Ala	22.5	22.0	22.8	18.5	14.7
f-mocPhe	21.0	21.7	18.4	16.2	18.5
f-moc Leu	19.3	21.2	17.7	16.1	17.6
f-moc Met	21.3	21.7	19.1	15.6	19.2
f-moc Pro	20.7	20.4	21.4	17.0	19.3
f-mocTrp	17.3	19.4	17.6	14.6	20.5

Mobile phase: 45% acetonitrile, buffer pH 2.5, concentration surfactant above CMC (15 mM). For PPEC: polarization voltage 800 V, experiment time - 10 min.

We saw that in both techniques, the surfactant type affects separation selectivity. This effect is due to interactions of all components of the system (tested substances, micelles and both the mobile and stationary phases). Regarding the HPTLC technique, the cationic surfactant of the longer hydrocarboneous chain (CTAB) enhances the solute retention, in comparison to the counterpart of this in the shorter chain (CTMA).This is the result of the CTAB system holding higher hydrophobicity in comparison to that with CTMA, due to the mentioned interaction (adsorption) of the polar head of cationic surfactant onto the negatively charged silanol group of the sorbent. Thus, mixed non-polar sorbent with C-18 and C-16 (from CTAB) hydrocarboneous chains is formed. Such mixed adsorbent brings about stronger retention of hydrophobic micelles containing f-moc amino acids. When CTMA is used, the above-mentioned interaction happens, but due to the C-1 group in CTMA, this affects only the free silanol group in the sorbent and does not protrude over on to the C-18 chain. Consequently, the hydrophobic interaction between the solute/CTMA micelle and the stationary phase is weaker, in comparison to the system containing CTAB. Nevertheless, the application of cationic surfactant produces little improvement in solute zone separation. Of note, the zones of Phe, Leu, Trp f-moc derivatives are not separated when the systems with the cationic surfactant are used.

Bearing in mind that the stationary phase interaction of anionic surfactants (sodium cholate and SDS) are only via hydrophobic interactions with the C-18 chain of sorbent, this result in changing the physicochemical character into that which is more hydrophilic. The bulky nonpolar cyclopenta[a]phenanthren group of sodium cholate additionally limits the specific interaction between sorbent and complex micelle–solute, and deteriorates the selectivity of solute separation (of note, the majority of f-moc derivatives are not, in fact, separated). In contrast, the flatter SDS molecule acts less obstructively on the above-mentioned specific interaction and engenders a better resolution of the solute zones. It also must be underlined that the solute retention in eluents containing SDS are more dissimilar.

Regarding the third surfactant type, non-ionic Brij-35, all possible interactions between all components of the system are naturally hydrophobic. Thus, this amfifile induces insignificant changes in the sorbent character. Consequently, a mobile phase inclusive of Brij-35 enhances retention of aliphatic and hydrophilic derivatives (f-moc Ala), while lessening the effect of more hydrophobic f-moc amino acids (Pro, Phe and Trp). Therefore, the zone separation is better than that for an eluent with sodium cholate, but poorer in comparison with that including SDS or cationic surfactants.

In assessing solute migration distances, we noted that the order of an amphiphile inclusive system is as follows (longest to shortest): Brij-35 and CTMA > SDS > sodium cholate > CTAB. For the majority of tensides, this sequence is in accordance with increasing CMC values for aqueous surfactant solutions as presented at the beginning of this section. The exception is CTAB, while for CTMA, there is no such data.

As for PPEC, cationic surfactants (CTAB or CTMA) in the mobile phase shorten the solute migration distances, since in applied electromigrational techniques (i.e. capillary electrophoresis), they create sorbents more hydrophobic and/or according to literature, reverse the electroosmotic flow direction^21^. Furthermore, in PPEC, cationic surfactants reduce the flow of the mobile phase^22^. We also found that a system containing CTAB is unable to separate f-moc Met and f-moc Pro, and a mobile phase with CTMA cannot distinguish between f-moc Phe and f-moc Leu. Interestingly, the longest migration distance in the CTAB-doped eluent is that of f-moc Trp, while the shortest is that of f-moc Ala. Such behaviour is contrary to other surfactant included mobile phases.

We discovered that eluents containing anionic or non-ionic surfactant (SDS, sodium cholate and Brij-35) generate considerably longer solute migration distances (SDS > Brij-35 > sodium cholate > CTAB > CTMA). Regarding anionic surfactants (SDS), literature data states that these have positive outcomes on the PPEC mobile phase flow^23^. This leads to longer solute migration distances, and the SDS-doped eluents separate all investigated f-moc derivatives quite well. In contrast, sodium cholate produces a solute separation that is similar that in HPTLC (poor separation of f-moc Phe, f-moc Leu and f-moc Met), while Brij-35 inclusion results in similar migration distances as that of sodium cholate-doped systems, as it has insignificant influence on electroosmotic flow. However, a mobile phase containing Brij-35 has poor solute zone characteristics, and, consequently, f-moc Phe and f-moc Met zones are not separated.

Thus the PPEC technique may be applied for systems with surfactant different than SDS. It is additionally resulting in changing solute selectivity.

### Statistical comparison of used techniques

Two f-moc amino acid derivatives(f-moc Ala and f-moc Trp) were chosen for statistical comparison of TLC and PPEC. The results are presented in Table 4.

**Table 4:** Statistical comparison of HPTLC and PPEC techniques for f-moc Ala and f-moc Trp.

	f-moc Ala		f-mocTrp	
	TLC	PPEC	TLC	PPEC
No	8	8	8	8
Average migration distance [mm]	17.95	32.21	12.62	22.19
median	17.80	31.98	12.65	22.08
variance	0.06	0.72	0.04	0.59
Standard deviation	0.24	0.79	0.19	0.74
% RSD	1.4	2.4	1.5	2.2

The mobile phase: 40% acetonitrile, 15 mM SDS, aqueous universal buffer of pH 2.5. For PPEC: polarisation voltage 1.5 kV, experiment time -15 min. HPTLC experiment time - 17 min.

As indicated in our results, and in accordance with literature data, the migration distances of the investigated amino acid derivatives are longer in PPEC, than in TLC^19,22–24^. However, after standard deviation and % RDS of results assessment, the above-mentioned technique is less reproducible. This phenomenon is characteristic of all electromigrational techniques.

### Effect of surfactant presence on separation efficiency

The mixture separation process in liquid chromatography is the result of a solute partition between stationary and mobile phases. During it, the solute zones undergo a dispersion process observable on the chromatogram or electrochromatogram as peak broadening or tailing. Both effects have an effect on separation efficiency. The presence of surfactant in the mobile phase influences the latter process. In the mobile phase, the influence is the result of mass transfer kinetics during the separation process^25^. This effect in liquid chromatography systems has been investigated in^26,27^. In these works, the authors related peak asymmetry, measured as left and right peak half-widths, to the separation system efficiency. However, these papers cover only HPLC-micellar systems. Since the aforementioned parameters have influence on peak broadening (asymmetry factor, A_s_) and peak tailing (peak tailing factor, T_f_), thus to the derivation of data, we decided to compare the effect of surfactants on TLC and PPEC, using the model compounds: f-moc Ala and f-moc –Trp. The results are seen in Table 5.

**Table 5:** Comparison of the surfactant effect on some chromatographic parameters in TLC and PPEC.

	Without SDS				With SDS (15 mM)			
	f-moc Ala		f-moc Trp		f-moc Ala		f-moc Trp	
	TLC	PPEC	TLC	PPEC	TLC	PPEC	TLC	PPEC
Migration distance (mm)	12.23	22.70	8.37	18.10	17.95	32.21	12.62	22.19
Peak asymmetry factor, A_s_*	1.52	1.29	2.17	1.23	0.98	0.98	1.26	1.04
Peak tailings factor, T_f_**	1.26	1.15	3.33	1.29	0.99	0.99	1.13	1.02
Separation efficiency, H_obs_ ***(mm)	0.097	0.074	0.080	0.055	0.092	0.058	0.078	0.077

Model solutes: f-moc Ala and f-moc Trp. The mobile phase: 40% acetonitrile, the aqueous universal buffer of pH 2.5. PPEC: polarisation voltage 1.5 kV, experiment time -15 min. TLC experiment time - 17 min.

*Peak asymmetry factor was calculated using the following equation: T_f_ = b/a, where b is the distance from the peak midpoint (perpendicular from the peak highest point) to the trailing edge of the peak measured at 10% of peak height (left peak half-width) and a is the distance from the leading edge of the peak to the peak midpoint (perpendicular from the peak highest point) to the trailing edge of the peak measured at 10% of peak height (right peak half-width).

**Peak tailing factor was calculated using the following equation: A_s_ = (a + b)/2a, where b is the distance from the peak midpoint (perpendicular from the peak highest point) to the trailing edge of the peak measured at 10% of peak height (left peak half-width) and a is the distance from the leading edge of the peak to the peak midpoint (perpendicular from the peak highest point) to the trailing edge of the peak measured at 10% of peak height (right peak half-width).

Both peak tailing and asymmetry factor formulas were found in^30^.

***Separation efficiency, the height of the theoretical plate, has been calculated using the following equation: ${H}_{obs}=\frac{{\sigma }^{2}}{{Z}_{x}}$

Where σ is the half of peak width at 0.607 height and Z_x_ is solute zone migration distance^30^.

For the system without surfactant, in TLC, both peak parameters (A_S_ and T_f_) for f-moc Ala and f-moc Trp significantly deviate from 1.0. Such behaviour indicates that mass transfer kinetics is disrupted. This effect affects peak broadening and/or tailing. Herein, solute interactions with the stationary phase have the essential effect that the solute of shorter migration distance (f-moc Trp) exhibits considerably higher A_s_ and T_f_ values when compared to the solute of longer migration distance (f-moc Ala). In PPEC without surfactant, A_s_ and T_f_ values are significantly less when compared to the TLC system results. Similarly, peak asymmetry and peak tailing factors are higher for the solute which migrates a shorter distance (f-moc Trp). On comparing separation efficiencies (H) for TLC and PPEC, the latter technique is better. Such behaviour is in accordance with literature data^19,22^.

The introduction of surfactant to the mobile phase significantly changes the property of the chromatographic system. In the TLC system and solute of shorter migration distance (f-moc Trp), A_s_ and T_f_ factors are lower when compared to the system without SDS. Moreover, for f-moc Trp, the deviation is close to 1.0. A similar effect is observed for f-moc Ala. In the PPEC technique, the inclusion of a surfactant in the eluent generated A_s_ and T_f_ values for the investigated f-moc derivatives that are close to ideal (1.0). Thus, the presence of SDS improves the shape of solute zones in both TLC and PPEC.

**Figure 2:**
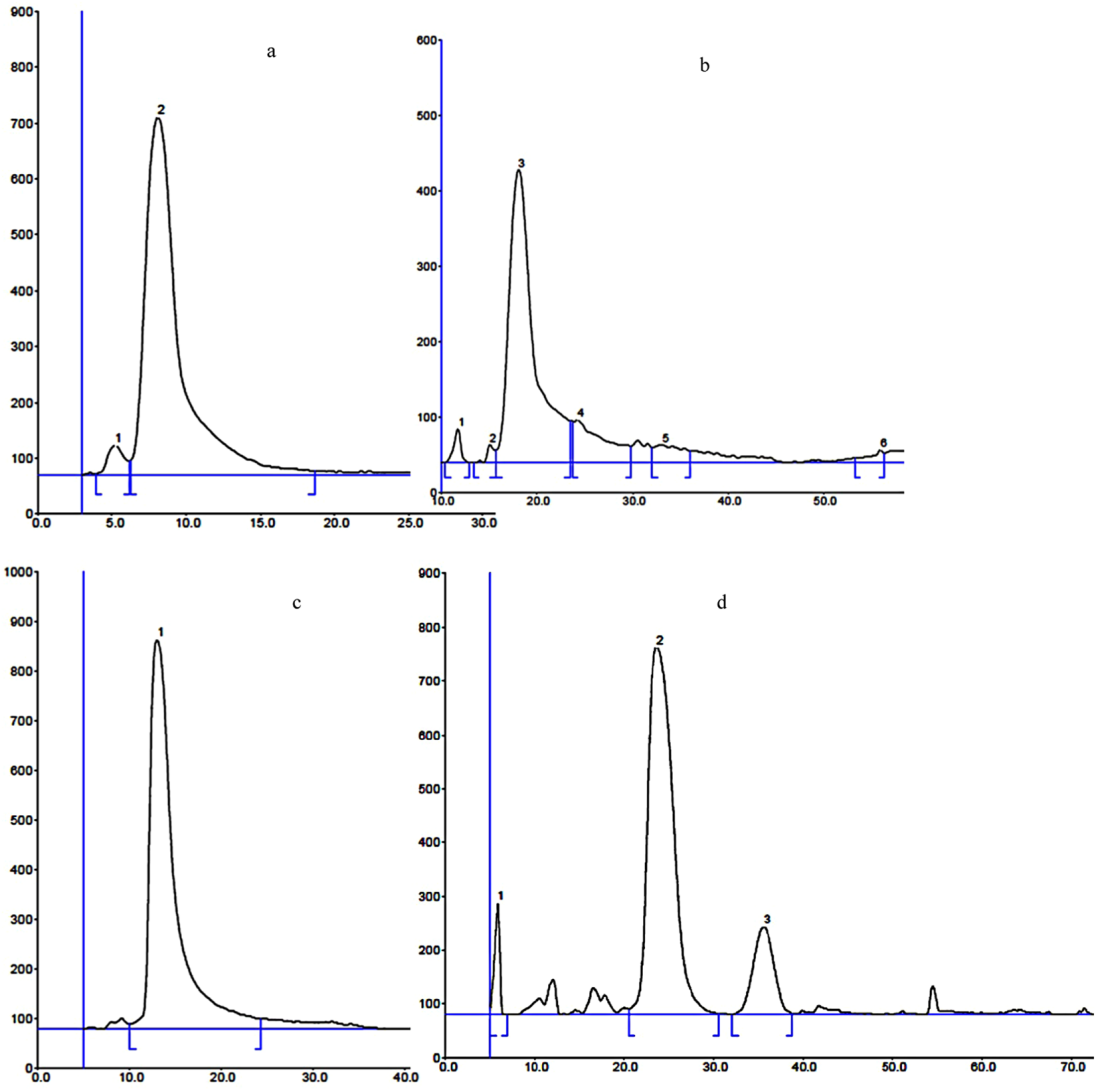
Separation of f-moc Trp with the use of HPTLC (eluent without SDS – (**a**); eluent with SDS – (**c**)) and PPEC (eluent without SDS – (**c**); eluent with SDS – (**d**)). The mobile phase: 40% acetonitrile, aqueous universal buffer of pH 2.5 (5% v/v), system with surfactant 15 mM SDS. Polarisation voltage in PPEC: 1.5 kV, experiment time 15 min.

Hence, with regard to separation efficiencies (H_obs_ values) for mobile phases containing surfactant, for both TLC and PPEC, the results are considerably better compared than eluent without SDS. However, the higher efficiency for f-moc Trp in a system without amphiphile and the effect of this on PPEC is worth further considering. As a general trend, the efficiencies for PPEC technique inclusive of eluent containing SDS are higher than that for TLC. The visual confirmation of above-mentioned thesis is presented on Fig. 2a–d. These Figs present densitograms (chromatograms and electrochromatograms) of the f-moc Trp detected at UV light of wavelength 262 nm. The zone shapes are better in PPEC technique when comparing to TLC one, especially for the mobile phase with surfactant (Fig. 2d) for which the peak is almost ideal.

### Test mixture separation

**Figure 3:**
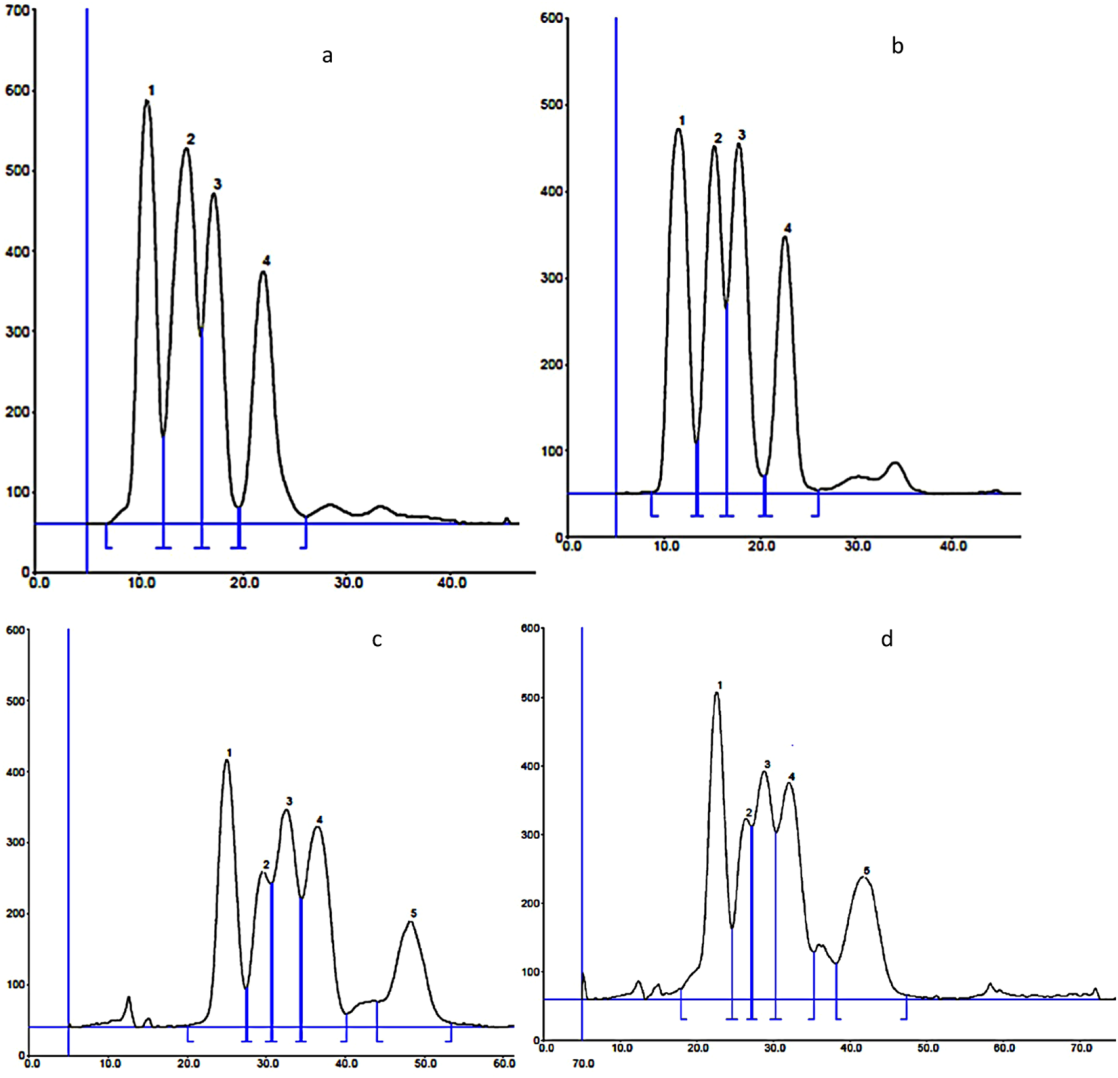
Separation of test mixtures with the use of HPTLC (mixture 1 – (**a**); mixture 2 - (**b**)) and PPEC (mixture 1 – (**c**); mixture 2 – (**d**)). The mobile phase: 40% acetonitrile, aqueous universal buffer of pH 2.5 (5% v/v), 15 mM SDS. Polarisation voltage in PPEC: 1.5 kV, experiment time 15 min. The order for mixture 1 in HPTLC: 1 - f-moc Trp + f- moc – Met; 2 - f-moc Pro; 3- f-moc Ala; 4- f-moc Cit; in PPEC: 1- f-moc Trp; 2 - f-moc Met; 3- f-moc Pro; 4 – f-moc Ala; 5- f-moc Cit. The order for mixture 2 in HPTLC: 1 - f-moc Met; 2- f- moc –Phe + f-moc Pro; 3- f-moc Ala; 4- f-moc Cit; in PPEC: 1- f-moc Phe; 2- f-moc Pro; 3- f-moc Leu; 4 – f-moc Ala; 5- f-moc Cit.

On the basis of the previous results for the separation of test mixtures, the system containing SDS and buffer of pH 2.5 was chosen for further investigation. Here, the remaining components of the mobile phase are 40% acetonitrile and 15 mM of SDS. To improve mixture separation during the PPEC experiments, 1.5 kV of polarization voltage was applied. The experiment time in both techniques lasted 15 min. Two test mixtures of f-moc amino acids were prepared. Mixture 1 contained f-moc derivatives of alanine, methionine, proline, tryptophan and citrulline (which was not investigated earlier; this amino acid derivative is more polar at log P = 1.95 (MervinSketch), in comparison with the other f-mocs). Mixture No 2 consisted of f-moc Ala, f-moc Leu, f-moc Phe, f-moc Pro and f-moc Cit. In the experiment, mixtures of the volume 4 μL were applied onto the sorbent with the use of ATS-4 applicator. The resulting chromatograms (TLC) and electrochromatograms (PPEC) are presented in Fig. 3a–d. The amino acid derivative zones were detected with the use of TLC scanner at 262 nm.

As indicated, the solute migration distances in PPEC are considerably longer in comparison with those from HPTLC. Additionally, the solute zones in HPTLC are quite well separated (compare Fig. 3a,c,b,d). What is more, calculating the peak resolution (R_s_) value for both investigated techniques and two last solute zones, the higher values we obtained for PPEC technique. Considering TLC technique (Fig. 3a,b) and peaks 3 and 4 for both mixtures, the R_s_ values are equal to 1.28 and 1.18, respectively. While for PPEC (Fig. 3c,d) peaks 4 and 5 for both above-mentioned mixtures the R_s_ values are 1.49 and 1.42, accordingly The order of separated solutes is in accordance with their hydrophobicity decrease.

## Conclusions

As a result of our experiments, we noted that the kind of surfactant applied and its concentration in the mobile phase has an influence on the f-moc amino acid migration distances in both HPTLC and PPEC. In HPTLC and PPEC experiments, eluents containing anionic surfactants (SDS or sodium cholate) are more convenient to use regarding the solute migration distances or retentions, in comparison with eluents containing cationic surfactants (CTAB, CTMA). This is because the latter have weaker interactions between the surfactant and the stationary phase. We also found that the concentration of surfactant in the eluent is a crucial factor affecting solute migration distances and the order of f-moc amino acids in both investigated techniques. In addition, depending on the technique used, the effect of the mobile phase buffer pH on retention, migration distance and zone separationof solutes vary. In HPTLC, the pH has insignificant influence on the solute retention of the majority solutes but affects the solute zone separations. In PPEC, pH affects both the solute migration distances and zone separations. Application of the PPEC technique also leads to significant improvement of the solute zone separations. We noted that the presence of surfactant in the mobile phase in the HPTLC technique results in two eluent migration fronts. Such a problem is eliminated in PPEC due to the pre-wetting process. Regarding a statistical comparison of these techniques, standard deviation and % RDS of the results indicate that PPEC is less reproducible. Still, introducing surfactant to the eluent results in a diminution of peak broadening in both techniques. What is more, the peak asymmetry and tailing factor values are lower for PPEC, in comparison to that of HPTLC. Of note, the efficiencies of separation of HPTLC are worse than that of PPEC. Therefore, due to its benefits, PPEC can be successfully applied, together with a micellar mobile phase, to separate mixtures.

## Methods

### Instrumentation

Prototype PPEC equipment was constructed in the Department of Physical Chemistry, Medical University of Lublin (Lublin, Poland). Its details are described in^24^. Horizontal DS-II-10 × 10 chambers for TLC were purchased from CHROMDES (Lublin, Poland). The automatic sampler ATS-4 and TLC scanner were provided by CAMAG (Muttenz, Switzerland).

### Materials

All investigated f-moc derivatives of amino acids (alanine, phenylalanine, leucine methionine, proline and tryptophan)were purchased from Sigma-Aldrich (Steinheim, Germany). Stock solutions containing 1 mg of each f-moc derivatives in 1 mL in acetone (extra pure, Avantor Performance Materials Poland SA, Gliwice, Poland) were prepared.

The structures and some physico-chemical properties of the investigated solutes such as pK_A_ (negative decimal logarithm from dissociation constants in aqueous solution), logP (hydrophobicity described as decimal logarithm from octanol-water partition coefficient) and molecular weights (MW) are presented in Table 1.

The mobile phases were hand-made by mixing various concentration of acetonitrile (extra pure, Avantor Performance Materials Poland SA, Gliwice, Poland) with buffer solution and surfactant. Two kinds of mobile phase buffers were used. The first (pH 4.25) consists of a mixture of sodium acetate (Avantor Performance Materials Poland SA, Gliwice, Poland) and acetic acid (Avantor Performance Materials Poland SA, Gliwice, Poland) aqueous solutions of 0.2 M. The second (universal) is comprised of citric acid (Merck, Darmstadt, Germany), Tris (tris (hydroxymethyl) aminomethane) (Merck, Darmstadt, Germany) and glycine (Merck, Darmstadt, Germany) all in concentration 0.008 M. The basic solution was either of two types. The first was titrated with a 3M KOH solution to obtain a pH of 10 (component A), whilst the second was titrated with a 1 M HCl solution until the pH was 2.5 (component B). The pH of both solutions was measured with a pH-meter CP 551 (Elmetron, Zabrze, Poland). Buffers used during the experiments were created by mixing the two above-mentioned components in appropriate proportions for pH 7.0 of B and A, in the ratio 50.6: 49.4, in accordance with^31^.

Sodium dodecyl sulphate, Brij-35, sodium cholate, hexadecyltriammonium bromide, and tetramethylammonium chloride were purchased from Merck (Darmstadt, Germany). The redistilled water used for preparation of the mobile phases was self-created.

Chromatographic plates, HPTLC RP18 W, 10 × 20 cm were purchased from Merck (Darmstadt, Germany).

The impregnates Sarsil W, Sarsil H50 and W hardener were purchased from Silikony Polskie (Nowa Sarzyna, Poland).

### Preparation of chromatographic plates

Commercially available HPTLC RP-18W plates (10 × 20 cm) were cut using a chromatographic plate cutter to a size of 10 × 10 cm. For the PPEC experiments, the plate edges were impregnated with a solution of Sarsil W and hardener (ratio of 96:4, one layer) and, subsequently, with a solution of Sarsil H-50 and hardener (ratio of 96:4; three layers). The painted margins were about 5 mm of width. After applying all layers, the plates were dried in an oven at 100 °C for 60 min, and were then transferred to a desiccator for cooling. Next, they were washed with methanol in a horizontal DS-chamber. After the methanol evaporated, the plates were activated in the drying chamber at 100 °C for 15 minutes, following which they were placed again into the desiccator. The plates for the TLC experiments were cleaned with methanol, activated in the drying chamber at 100 °C for 15 minutes and placed into the desiccator.

### Application of samples

For the PPEC experiments, 2 µL of each f-moc amino acid derivative solution was applied 15 mm from the lower edge of the previously prepared electrochromatographic plate, at a zone with a length of 6 mm, utilizing the automatic sampler ATS4. The spot application began 15 mm from the right edge and finished 15 mm from the left edge of the plate. For the TLC experiments, the above-mentioned volume of each solution of f-moc amino acid derivative was applied 5 mm from the lower edge of the previously prepared chromatographic plates. The remaining preparation practices were similar as that in PPEC.

### Development of chromatograms and electrochromatograms

Chromatograms were developed at room temperature, using a Horizontal DS Chamber for TLC, model DS-II–10 × 10. The sorbent layer was first equilibrated by standing 15 min inside the mobile phase vapour before the plate development. The distance of the eluent was 45 mm from the application line. The development of electrochromatograms using PPEC equipment is described in^24^. Before development, the electrochromatographic plates were prewetted for 1 min with the mobile phase. The polarization voltage used varied from 800 to 1500 V depending on the experiment. Consequently, PPEC experiment times were also distinct.

### Solutes detection

F-moc amino acids zones on the chromatographic and electrochromatographic plates were identified with the use of TLC scanner (CAMAG, Muttenz, Switzerland) at 262 nm.

### Ethical approval

This article does not contain any studies with human participants or animals performed by any of the authors.
